# Supplementary material for: Acute kidney injury in Ugandan children with severe malaria is associated with long-term behavioral problems
Source: PLoS One. 2019 Dec 17;14(12):e0226405. doi: 10.1371/journal.pone.0226405 (PMC6917349; doi:10.1371/journal.pone.0226405)
Supplement: S1 Table — (DOCX) [file pone.0226405.s002.docx]

**S1 Table. Primary behavioral outcomes for community children based on the presence of malaria by PCR on enrollment**

|  | Unadjusted Estimates | | | Adjusted Estimates | | |
| --- | --- | --- | --- | --- | --- | --- |
|  | N (obs.), N | Coefficient (95% CI) | *P* | N (obs.), N | Coefficient (95% CI) | *P* |
| Children aged <6 years |  |  |  |  |  |  |
| Socio-emotional function^a^ | | | | | | |
| Internalizing behavior | 511, 148 | 0.35 (0.10, 0.60) | 0.006 | 510, 147 | 0.04 (-0.20, 0.28) | 0.76 |
| Externalizing behavior | 511, 148 | 0.21 (-0.11, 0.52) | 0.20 | 510, 147 | 0.06 (-0.27, 0.39) | 0.71 |
| Executive function^b^ | | | | | | |
| Global Executive Composite | 344, 119 | 0.41 (-0.06, 0.88) | 0.08 | 273, 118 | 0.10 (-0.37, 0.56) | 0.68 |
| Emergent Metacognition Index | 344, 119 | 0.26 (-0.20, 0.71) | 0.27 | 273, 118 | -0.02 (-0.51, 0.48) | 0.95 |
| Flexibility Index | 344, 119 | 0.36 (-0.07, 0.78) | 0.10 | 273, 118 | -0.02 (-0.42, 0.37) | 0.90 |
| Inhibitory Self-Control Index | 344, 119 | 0.50 (0.003, 0.99) | 0.05 | 273, 118 | 0.20 (-0.29, 0.68) | 0.43 |
| Children aged ≥6 years |  |  |  |  |  |  |
| Socio-emotional function^a^ | | | | | | |
| Internalizing behavior | 137, 63 | 0.28 (-0.13, 0.69) | 0.18 | 137, 63 | 0.21 (-0.10, 0.52) | 0.18 |
| Externalizing behavior | 137, 63 | 0.20 (-0.27, 0.68) | 0.40 | 137, 63 | -0.13 (-0.62, 0.36) | 0.60 |
| Executive function^b^ | | | | | | |
| Global Executive Composite | 87, 60 | 0.30 (-0.14, 0.74) | 0.18 | 87, 60 | -0.08 (-0.54, 0.37) | 0.72 |
| Emergent Metacognition Index | 87, 60 | 0.23 (-0.16, 0.62) | 0.24 | 87, 60 | -0.17 (-0.57, 0.23) | 0.40 |
| Behavior Regulation Index | 87, 60 | 0.31 (-0.21, 0.83) | 0.23 | 87, 60 | 0.07 (-0.52, 0.65) | 0.82 |

**Abbreviations:** CI, confidence interval; P, P-value; N (obs.), number of observations in the model; N, the number of children in the analysis.

^a^Assessed using the Child Behavior Checklist (CBCL)

^b^Assessed using the Behavior Rating Inventory of Executive Function (BRIEF)

All linear mixed models were fitted with a subject specific random intercept and a caretaker random effect (for children <6 years of age) and visit as a categorical variable (baseline, 6 months, 12 months, 24 months). Adjusted models included age, sex, height-for-age, weight-for-age, socioeconomic status, home environment, maternal education, preschool exposure, year of enrollment and test administrator as fixed effects.
