# Supplementary material for: Acute kidney injury in Ugandan children with severe malaria is associated with long-term behavioral problems
Source: PLoS One. 2019 Dec 17;14(12):e0226405. doi: 10.1371/journal.pone.0226405 (PMC6917349; doi:10.1371/journal.pone.0226405)
Supplement: S2 Table — (DOCX) [file pone.0226405.s003.docx]

**S2 Table. Secondary socio-emotional outcomes for children** <**6 years with severe malaria by acute kidney injury (AKI) status**

|  | Unadjusted models | | | Adjusted Models | | |
| --- | --- | --- | --- | --- | --- | --- |
|  | N (obs.), N | Mean difference (95% CI)  AKI vs. No AKI | *P* | N (obs.), N | Mean difference (95% CI)  AKI vs. No AKI | *P* |
| **Socio-emotional function^a^** | | | | | | |
| Internalizing scales |  |  |  |  |  |  |
| Anxious/depressed | 1296, 379 | 0.03 (-0.12, 0.18) | 0.71 | 1276, 370 | -0.09 (-0.23, 0.06) | 0.24 |
| Withdraw/depressed | 1296, 379 | 0.20 (0.02, 0.37) | 0.03 | 1276, 370 | 0.03 (-0.14, 0.21) | 0.71 |
| Somatic complaints | 1296, 379 | 0.04 (-0.12, 0.19) | 0.63 | 1276, 370 | -0.13 (-0.28, 0.02) | 0.09 |
| Emotionally reactive | 1296, 379 | 0.03 (-0.12, 0.19) | 0.69 | 1276, 370 | -0.07 (-0.23, 0.09) | 0.39 |
| Externalizing scales |  |  |  |  |  |  |
| Aggressive behavior | 1296, 379 | 0.18 (0.01, 0.36) | 0.04 | 1276, 370 | 0.04 (-0.14, 0.22) | 0.69 |
| Attention problems | 1296, 379 | 0.13 (-0.0003, 0.26) | 0.05 | 1276, 370 | 0.10 (-0.04, 0.24) | 0.16 |
| Sleep problems | 1296, 379 | 0.10 (-0.07, 0.27) | 0.23 | 1276, 370 | -0.05 (-0.23, 0.14) | 0.62 |
| **Executive function^b^** | | | | | | |
| Behavior Regulation scales |  |  |  |  |  |  |
| Inhibit | 726, 298 | 0.34 (0.06, 0.63) | 0.02 | 571, 283 | 0.03 (-0.28, 0.34) | 0.86 |
| Task shifting | 726, 298 | 0.33 (0.09, 0.57) | 0.008 | 571, 283 | 0.08 (-0.16, 0.32) | 0.52 |
| Emotional control | 726, 298 | 0.14 (-0.09, 0.37) | 0.23 | 571, 283 | -0.16 (-0.41, 0.08) | 0.18 |
| Metacognition Index |  |  |  |  |  |  |
| Plan/organize | 726, 298 | 0.47 (0.18, 0.75) | 0.001 | 571, 283 | 0.10 (-0.18, 0.38) | 0.49 |
| Working memory | 726, 298 | 0.43 (0.18, 0.69) | 0.001 | 571, 283 | 0.16 (-0.11, 0.43) | 0.25 |

**Abbreviations:** CI, confidence interval; P, P-value; N (obs.), number of observations in the model; N, the number of children in the analysis.

^a^Assessed using the Child Behavior Checklist (CBCL)

^b^Assessed using the Behavior Rating Inventory of Executive Function (BRIEF)

All linear mixed models were fitted with a subject specific random intercept and a caretaker random effect and visit as a categorical variable (baseline, 6 months, 12 months, 24 months).

Adjusted models included age, sex, height-for-age, weight-for-age, socioeconomic status, home environment, maternal education, preschool exposure, presence of coma on admission, number of seizures during hospitalization, parenteral antimalarial treatment (quinine vs. artemisinin), year of enrollment and test administrator as fixed effects.
