# Supplementary material for: Acute kidney injury in Ugandan children with severe malaria is associated with long-term behavioral problems
Source: PLoS One. 2019 Dec 17;14(12):e0226405. doi: 10.1371/journal.pone.0226405 (PMC6917349; doi:10.1371/journal.pone.0226405)
Supplement: S4 Table — (DOCX) [file pone.0226405.s005.docx]

**S4 Table. Behavioral outcomes for children ≥6 years with severe malaria by acute kidney injury (AKI) status stratified by study group at enrollment**

|  | Cerebral Malaria | | | Severe Malarial Anemia | | |
| --- | --- | --- | --- | --- | --- | --- |
|  | N (obs.), N | Coefficient (95% CI) | *P* | N (obs.), N | Coefficient (95% CI) | *P* |
| Socio-emotional function^a^ | | | | | | |
| Internalizing behavior | 219, 84 | 0.17 (-0.14, 0.48) | 0.280 | 138, 65 | -0.22 (-0.62, 0.18) | 0.280 |
| Externalizing behavior | 219, 84 | **0.61 (0.13, 1.09)** | **0.013** | 138, 65 | **0.36 (-0.18, 0.89)** | **0.190** |
| Executive function^b^ | | | | | | |
| Global Executive Composite | 155, 79 | 0.58 (0.10, 1.05) | 0.019 | 96, 58 | **0.64 (0.04, 1.24)** | **0.036** |
| Emergent Metacognition Index | 155, 79 | 0.37 (-0.13, 0.89) | 0.148 | 96, 58 | 0.48 (-0.16, 1.12) | 0.140 |
| Behavior Regulation Index | 155, 79 | **0.73 (0.28, 1.19)** | **0.002** | 96, 58 | **0.71 (0.07, 1.35)** | **0.031** |

**Abbreviations:** CI, confidence interval; P, P-value; N (obs.), number of observations in the model; N, the number of children in the analysis.

^a^Assessed using the Child Behavior Checklist (CBCL)

^b^Assessed using the Behavior Rating Inventory of Executive Function (BRIEF)

All linear mixed models were fitted with a subject specific random intercept and visit as a categorical variable (baseline, 6 months, 12 months, 24 months).

Adjusted models included age, sex, height-for-age, weight-for-age, socioeconomic status, home environment, maternal education, preschool exposure, number of seizures during hospitalization (in cerebral malaria), parenteral antimalarial treatment (quinine vs. artemisinin), year of enrollment and test administrator as fixed effects.
