# Supplementary material for: Acute kidney injury in Ugandan children with severe malaria is associated with long-term behavioral problems
Source: PLoS One. 2019 Dec 17;14(12):e0226405. doi: 10.1371/journal.pone.0226405 (PMC6917349; doi:10.1371/journal.pone.0226405)
Supplement: Attachment — Submitted filename: PONE Response to Reviewers 2019.11.01.docx [file pone.0226405.s006.docx]

Dear Prof. Craig,

Thank you for your comments, and invitation to resubmit our manuscript. Please see our point-by-point response to the reviewer’s comments below, and the revised manuscript.

**Reviewers' comments:**

**Reviewer #1**: This is a report assessing the correlation between malaria associated Acute Kidney Injury (AKI) and long-term behavioral problems. The group is a leader in the malaria field and has recently clarified and highlighted the previously under-recognized role of kidney injury in pediatric malaria. The report uses a cohort of malaria patients in Uganda that have been used for multiple publications, including a recent report in BMC Medicine showing the effect of AKI on short- and long-term neuro cognitive outcomes. Using the same cohort, the investigators use the CBCL and BRIEF behavioral assessment tools for two years post discharge. They have found significant deficits in the areas of externalizing behavior, global executive function, and behavior regulation in those children with malaria-related AKI. These findings remain significant in a mixed model that includes twelve covariates as fixed effects.

The study is well performed, the data analyzed appropriately and the results clear. The problem is a large one – with malaria related morbidity having an immense impact across sub-Saharan Africa. I would recommend publication with only the following small comments.

**1) I am interested in the role of AKI itself (as opposed to AKI specifically in the setting of malaria) in these long-term sequelae. Are there any data on the role of AKI of other etiologies on long-term neurocognitive or behavioral changes?**

**Response:** To our knowledge, our data are the first to describe a relationship between AKI and long-term behavioral changes. We agree with the reviewer that studies are needed to evaluate whether AKI is related to similar long-term neurocognitive and behavioral changes in the context of critical illness, and this is something we hope to explore in the future.

We have added a sentence to the discussion highlighting the need for studies to assess long-term neurocognitive and behavioral outcomes in the context of AKI in hospitalized children. It states (page 16, lines 289-290), “Additional studies are needed to determine whether long-term neurocognitive and behavior problems associated with AKI are specific to severe malaria, or generalizable to other populations with critical illness.”

**2) The conclusion sentence may be confusing for those without a knowledge of the definition of ‘school aged children’. Perhaps the sentence should explicitly state the cut-off of 6 years of age.**

**Response:** Thank you for this comment. We have revised the sentence as suggested on page 2, line 36.

**3) Very minor. There appear to be some typos in the sentences starting on lines 115 and 277.**

**Response:** Thank you for pointing out these errors. We have made the appropriate edits to the revised manuscript.

**Reviewer #2:** This is a well conducted study in a fairly large number of African children which has expanded on the authors previous studies which evaluated the association between AKI and neurological disability and cognitive impairment. The authors have now detailed the impact of CM/SM and AKI on behavioral issues with longitudinal assessment. This finding done in a robust and standardized method is novel data.

**There are only a few minor comments
1) Was creatinine only assessed at enrollment? How did the authors assess if impaired renal function at the time of assessment at months 12 and 24 months contribute to their results.**

**Response:** We assessed creatinine at enrollment and 12 months follow-up where we defined chronic kidney disease (CKD) based on an eGFR<90mL/min per 1.73m^2^ as previously reported (Conroy et al., 2019 *BMC Med* ). We had 16 children with evidence of CKD at one year follow-up, and 7 of them had AKI during their original admission. When we stratified based on the child’s age at 12 months follow-up, we were underpowered to evaluate how CKD, and the interaction between CKD and AKI may impact behavioral outcomes. At 12 months follow-up, there were only two children with CKD in school age children where we observed the effects between AKI and behavior.

There are documented impacts of CKD on cognition in other pediatric populations, and this is an area of active interest in our research group. We are currently starting long-term follow-up studies of multiple cohorts of children with severe malaria to evaluate the prevalence of CKD and how it relates to health related quality of life, academic achievement, cognition, behavior and mental health.

We have included a statement in the discussion highlighting the need for follow-up studies to assess the impact of CKD on child development on page 16 lines 290-292, stating: “Further, as AKI is a risk factor for subsequent development of chronic kidney disease in children [28, 43, 44], follow-up studies are needed to delineate the long-term impact of chronic kidney disease on child development in survivors.”

**2) It will be good to get more details on who did the behavioral assessments and how were they trained to ensure standardization**

**Response:** The behavioral assessments were conducted by trained research assistants with a Bachelor’s degrees in psychology, and all tests were conducted according to standard operating procedures. Training was conducted by a graduate level psychologist or experienced research assistants who scored the same interviews as the research assistant, and testers were allowed to test study participants once there was no discrepancy in the assessments between trainers and testers. Testing was subjected to a continuous quality control program by a quality control officer that provided feedback on the test assessments to ensure standardization. As the tests were conducted over a number of years and there were different testers that conducted the evaluations over time, we adjusted for both time and neuropsychological tester in our analysis.

We have added additional details to the Methods related to training and standardization of the neuropsychological testing on page 5, lines 104-107. The section now states “Behavioral assessments were conducted according to standard operating procedures by trained research assistants with a Bachelor’s degrees in psychology. Testing was subjected to a continuous quality control program by a quality control officer that provided feedback on the tests to ensure standardization.”
